# Editorial: Vaccine and infectious disease informatics

**DOI:** 10.3389/fcimb.2026.1977304

**Published:** 2026-09-18

**Authors:** Junguk Hur, Zhiwei Cao, Nikolai Petrovsky, Vladimir Brusic, Cui Tao, Yongqun He

**Affiliations:** 1 Department of Biomedical Sciences, University of North Dakota, Grand Forks, ND, United States; 2 School of Life Sciences, Fudan University, Shanghai, China; 3 Australian Respiratory and Sleep Medicine Institute Ltd, Adelaide, SA, Australia; 4 School of Rural Medicine, University of New England, Armidale, NSW, Australia; 5 Vaxine Pty Ltd, Adelaide, SA, Australia; 6 Metropolitan College, Boston University, Boston, MA, United States; 7 Department of Artificial Intelligence and Informatics, Mayo Clinic in Florida, Jacksonville, FL, United States; 8 Unit for Laboratory Animal Medicine, Department of Microbiology and Immunology, and Center for Computational Medicine and Bioinformatics, University of Michigan Medical School, Ann Arbor, MI, United States

**Keywords:** bioinformatics, immunoinformatics, infectious disease, machine learning, ontology, reverse vaccinology, vaccine informatics, vaccine safety surveillance

## 1 Introduction

Infectious diseases remain a leading cause of death worldwide. COVID-19, with several million recorded deaths, was a stark reminder that controlling infections depends on vaccines that are both safe and effective (Collaborators, 2022). Designing such vaccines depends in turn on a mechanistic understanding of pathogenesis and protective immunity. COVID-19 was not an isolated case. Measles has resurged in under-vaccinated communities, monkey pox spread across multiple countries in 2022 and 2023, pertussis remains a major issue, drug-resistant tuberculosis is on the rise, and avian influenza strains such as H5N1 continue to circulate (Thornhill et al., 2022; Minta et al., 2024).

Today’s vaccine technologies differ fundamentally from those of the 1990s: the change has been structural, moving vaccinology from manipulating pathogens at the bench to digital, platform-based design (Rappuoli et al., 2016). Molecular immunology defined the biochemical rules of immune recognition, from peptide- Major Histocompatibility Complex (MHC) binding motifs to antibody-antigen structural determinants. Curated databases then made that knowledge computable, from pathogen genomes to the International ImMunoGeneTics information system (Giudicelli et al., 1997) and the Immune Epitope Database (Peters et al., 2005). Immunoinformatics supplied the predictive engines, from the quantitative matrices and neural networks of 1994 to today’s epitope predictors. These strands have since converged into integrated platforms that shift the goal from predicting targets to designing immunogens with broad population coverage.

What has shifted over the past decade is not only knowledge of biology, but the scale and heterogeneity of the data being generated. Omics technologies, electronic health records, clinical trial registries, and post-licensure reporting systems now generate more information than any laboratory can read by hand. Turning that flood of data into knowledge is the work of informatics: the standards, ontologies, knowledgebases, and algorithms that make heterogeneous data *findable, accessible, interoperable*, and *reusable* (FAIR) rather than merely abundant (Wilkinson et al., 2016).

We assembled this Research Topic “Vaccine and Infectious Disease Informatics” to capture progress where informatics, vaccinology, and infectious disease research meet. The 21 articles collected here, representing more than 150 authors and published in *Frontiers in Cellular and Infection Microbiology* and *Frontiers in Public Health*, run from literature mining and omics analysis to reverse and structural vaccinology, immunoinformatics, mathematical modeling, clinical prediction, and post-licensure safety surveillance. They fall into five groups, described below.

## 2 Informatics infrastructure: ontologies, knowledgebases, and standardized integration

One theme underlies the rest: infrastructure, the reusable scaffolding without which fragmented data cannot be compared. Three contributions illustrate this theme.

Monickaraj et al. present Vaxjo 2.0, an ontology- and large language model (LLM)-powered knowledgebase of vaccine adjuvants that grows coverage from 103 to 448 adjuvants standardized in the Vaccine Ontology (Zheng et al., 2025) across 16 categories. A two-phase LLM pipeline mined 817 PubMed abstracts to annotate mechanisms for 323 adjuvants across 16 mechanism families. In a 50-adjuvant sample, it reached 82% agreement with expert annotation, covering 146 of 178 mechanism subtypes. The key contribution is this pairing: the ontology supplies controlled vocabulary, the language model supplies scale, and human curation provides quality control.

Huffman et al. extend the VIOLIN knowledgebase with 2,847 experimentally verified viral vaccines against 95 species, then turn reverse vaccinology tools (Vaxign2 and Vaxign-ML) back on the data to show that protective antigens are markedly more protegenic than non-protective ones (88.0 versus 79.5). They also demonstrate that structural surface proteins make better candidates, supporting the use of a protegenicity cutoff near 85% in that analysis.

Wang et al. apply the same standardizing logic to the host rather than the vaccine, running an Ontology-based Evidence Synthesis and Reasoning (O-ESR) pipeline on the Ontology of Human-Microbiome Interactions (OHMI) framework to reconcile chronic obstructive pulmonary disease microbiome findings from more than 30 studies. Automated reasoning over more than 100 altered taxa recovers a coherent gut-lung axis, with pathogenic Proteobacteria expanding in the airway as short-chain-fatty-acid producers thin out in the gut. Across the three, the shared aim is to turn scattered observations into computationally queryable knowledge.

## 3 Computational prediction, design, and discovery

Where the first group of papers standardizes existing knowledge, the second group uses computation to predict what has not yet been observed.

Ye et al. describe a training-free, structure-based pipeline that ranks antibodies by conformational epitope, conserved-surface mapping, and immunogenicity similarity. The pipeline was run over 1,273 antibodies against five coronavirus spike antigens, including four SARS-CoV-2 variants and SARS-CoV. Its top-20 list placed 80% (16 of 20) on the predicted receptor-binding domain and confirmed 45% (9 of 20) as cross-reactive in CoV-AbDab, almost twice the hit rate of an established network-based method.

Imani et al. provide a broader review of the toolkit, examining how computational biology and artificial intelligence (AI) now thread neoantigen prediction, mRNA sequence optimization, and lipid-nanoparticle design into one workflow for personalized cancer vaccines, adapting tools proven at pandemic scale to oncology.

Llanes et al. evaluate *in silico*-predicted B-cell epitopes spanning the entire proteomes of *Anaplasma platys* and *Ehrlichia canis* using peptide microarrays and recover 1,220 immunoreactive peptides, 82 of them shared between the two pathogens. These findings provide a cost-effective route to diagnostic and vaccine antigens. Clinical prediction is represented by two studies.

Liu et al. develop a web-accessible, explainable risk model for pediatric varicella encephalitis from six routine variables, chosen by agreement among LASSO, XGBoost, and random forests. The model reports an area under the curve of 0.950 in their data.

Zhang et al. show that one inexpensive marker, admission serum ferritin, was independently associated with death and vasopressor use in elderly pneumonia and improved the discrimination of the Sequential Organ Failure Assessment (SOFA) and CURB-65 scores when added to them. What links molecular design to bedside triage here is less a shared method than a shared discipline: predictions transparent enough to be checked and reproduced.

## 4 Experimental vaccine development and immunogenicity

Three contributions move from data to the bench, each reporting a new vaccine candidate and the preclinical evidence supporting it.

Yang et al. report toxicity and immunogenicity studies for a Chinese hamster ovary (CHO) cell recombinant zoster vaccine built on varicella-zoster glycoprotein E and the new BC02 adjuvant, with no target-organ toxicity in rats and durable humoral and cellular responses, providing evidence to justify moving into the clinic.

Ren et al. took a mucosal route with an intranasal trivalent protein vaccine that raised broad systemic and mucosal immunity against pneumococcal pneumonia, cut colonization and lung injury after challenge, and outperformed its mono- and bivalent comparators on survival.

Wang et al. push vaccinology into One Health territory with a replication-defective adenovirus-vectored SARS-CoV-2 candidate that protected mice in a challenge model and reduced viral shedding in cats and minks, highlighting the relevance of animal reservoirs and cross-species transmission to vaccine development.

These experimental results detailed in the above papers provide the evidence base that computational design seeks to anticipate and that post-licensure surveillance later evaluates. They also define the immunogenicity, toxicity, protection, and shedding endpoints that informatics resources need to capture in reusable, comparable form.

## 5 Post-licensure vaccine safety surveillance

Once a vaccine is licensed, an additional question is how it behaves at the population scale. This is the Research Topic’s largest and, in the current climate, one of its most closely scrutinized and important topics. Six papers analyze the United States Vaccine Adverse Event Reporting System (VAERS) (Shimabukuro et al., 2015) using the standard disproportionality toolkit (proportional reporting ratio, reporting odds ratio, Bayesian confidence propagation neural network, and the multi-item gamma Poisson shrinker) (Bate et al., 1998; Rothman et al., 2004). They fall into three groups according to what their signals imply for practice.

The first three articles broadly reassure safety profiles for widely used vaccines. Liu et al. analyze more than 193,000 diphtheria-tetanus-acellular pertussis (Tdap) vaccine adverse event reports in children under seven and find the familiar pattern of overwhelmingly non-serious, early-onset events. Fan et al. examine two decades of standalone maternal Tdap immunization data, treating the prominent pregnancy-related signals as hypothesis-generating reporting artifacts consistent with background rates rather than evidence of harm, and interpret their results as consistent with the continued use of maternal Tdap. Chen et al. reach a similar verdict for the 9-valent human papillomavirus (HPV) vaccine over its first decade, where 92.5% of reports were non-serious, and no major new pattern departed from the pre-licensure profile.

The next two papers identify specific VAERS (Vaccine Adverse Event Reporting System) signals that warrant active follow-up. Wei et al. flag stronger hexavalent than pentavalent safety signals in the youngest infants, notably apnea and cyanosis. They stress, however, that both vaccines remain consistent with their established safety profiles and that the pattern should prompt active monitoring rather than be interpreted as causal. Zhou et al. report a disproportionality signal involving autoimmune thyroiditis and Graves disease with female predominance, for a combined hepatitis A and B vaccine. They caution that the signal requires confirmation in systems with denominator and comparator data.

The final paper maps the temporal landscape of VAERS reporting. Zheng et al. analyze 632,481 pneumococcal reports, most of which were non-serious and clustered within thirty days of vaccination. Their analysis defines temporal windows for post-licensure monitoring.

Across these papers, the same caution recurs: a disproportionality signal is a reporting association, useful for directing active surveillance but not for inferring causation. This Research Topic identifies the need for better monitoring and informatics systems to be developed for assessing vaccine safety ideally in real time, identifying VAERS as a cumbersome, incomplete, delayed and imprecise system of identifying potential safety signals, placing it at odds with modern day population monitoring and informatics technologies and capabilities.

## 6 The epidemiological and clinical evidence landscape

The last four papers widen the lens to systems of infectious disease care, policy, and the research record itself. Two papers focus on tetanus. Huang et al. trace adult generalized tetanus through a Chinese tertiary hospital, documenting that this wholly vaccine-preventable disease still falls hardest on older, rural, unvaccinated patients, is costly and often fatal, and that the systemic immune-inflammation index independently marks the most severe cases. Zhou et al. provide a two-decade bibliometric survey of tetanus vaccine and immunoglobulin research, charting the shift toward maternal immunization, lower-resource implementation, and the expanding role of tetanus toxoid as a conjugate carrier.

The two other papers turn to the systems that govern vaccines and to the public-health record itself. Zhong et al. examine the upstream evidence base, cataloging 620 vaccine trials on China’s national registry from 2013 to 2024 and documenting the measurable effect of the 2019 Vaccine Management Law on how trials are run and insured. Tahir et al. close with a sobering perspective on measles returning to the United States on the back of vaccine hesitancy, pandemic-disrupted immunization schedules, and travel, underscoring that measles elimination is secure only above 95% two-dose coverage.

Together, these papers emphasize that informatics has its greatest impact when its conclusions inform clinical practice, regulatory decision-making, and public-health communication.

## 7 Conclusions and future perspectives

The Research Topic traces an arc from standardized data, through prediction, design, and experimental proof, to post-licensure surveillance and public-health practice. Vaccine and infectious disease informatics is no longer mainly about database construction or antigen prediction, but a field spanning data standards, AI-assisted curation, predictive modeling, experimental validation, real-world evidence, and public-health surveillance, built on shared ontologies and knowledgebases. An important next step is to link these resources so that the Vaccine Ontology behind Vaxjo 2.0 and VIOLIN, the OHMI framework, and regulatory vocabularies such as MedDRA (Brown et al., 1999) can be queried together rather than in parallel.

Artificial intelligence, including large language models, has moved into practical use for data curation, antigen and antibody prediction, vaccine design, and clinical risk scoring. What it needs is governance: versioned models and prompts, validation sets with reported accuracy, and provenance separating model-generated suggestions from expert-verified assertions, so that knowledgebase entries remain auditable years later. Post-licensure surveillance remains a qualitative discipline whose credibility rests on the restraint these authors show in reading signals as associations rather than causes. A One Health perspective runs through several contributions, treating human, animal, and environmental health as one problem.

The limitations are equally clear. Much of the evidence is retrospective, single-center, or drawn from spontaneous reporting systems with unknown denominators and well-documented biases. Predictive models will ultimately be judged by prospective, multi-site validation, not retrospective discrimination. Consistent with the FAIR principles, future contributions should release datasets, model versions, code, and ontology mappings by default, so that results can be reproduced and extended, not only cited. We thank the authors, the reviewers, and the Frontiers staff for bringing this Research Topic to publication.
